# Flower visits and pollinator pollen load networks reveal the effects of pollinator sharing on heterospecific pollen transfer in a subalpine plant community

**DOI:** 10.1002/ece3.11244

**Published:** 2024-04-08

**Authors:** Qiang Fang, Jiajun Wu, Tao Zhang, Suyan Ba, Chunyan Zhao, Panfeng Dai

**Affiliations:** ^1^ College of Agriculture Henan University of Science and Technology Luoyang China; ^2^ Ministry of Education Key Laboratory for Transboundary Ecosecurity of Southwest China, Yunnan Key Laboratory of Plant Reproductive Adaptation and Evolutionary Ecology, Institute of Biodiversity, School of Ecology and Environmental Science Yunnan University Kunming Yunnan China

**Keywords:** heterospecific pollen transfer, indirect interactions, interspecific competition, plant–pollinator networks, pollinator pollen load, pollinator sharing

## Abstract

The mutualistic network of plant–pollinator also involves interspecific pollination caused by pollinator sharing. Plant–pollinator networks are commonly based on flower visit observations, which may not adequately represent the actual pollen transfer between co‐flowering plant species. Here, we compared the network structure of plant–pollinator interactions based on flower visits (FV) and pollen loads (PL) on the bodies of pollinators and tested how the degree of pollinator sharing in the two networks affected heterospecific pollen transfer (HPT) between plant species in a subalpine meadow. The FV and PL networks were largely overlapped. PL network included more links than FV network. The positions of plant and pollinator species in the FV and PL networks were positively correlated, indicating that both networks could detect major plant–pollinator interactions. The degree of pollinator sharing, based on either the FV or the PL network, positively influenced the amount of heterospecific pollen transferred between plant species pairs. However, the degree of pollinator sharing had a low overall explanatory power for HPT, and the explanatory powers of the FV and PL networks were similar. Overall, our study highlights the importance of FV and PL for understanding the drivers and outcomes of plant–pollinator interactions, as well as their relevance to HPT.

## Graphical abstract

The degree of pollinator sharing, based on either the flower visits or the pollinator pollen load networks, positively influenced the amount of heterospecific pollen transferred between plant species pairs. However, the degree of pollinator sharing had a low overall explanatory power for heterospecific pollen transfer, and the explanatory powers of the flower visit and pollinator pollen load networks were similar.

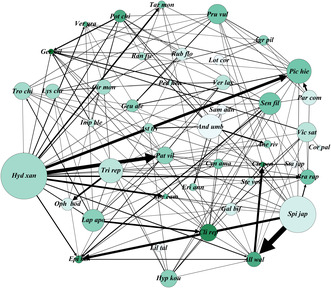

## 1 INTRODUCTION

Co‐flowering plant species that share pollinators may experience both mutualism and competition in interactions with pollinators (Arceo‐Gómez & Ashman, 2014; Fang et al., 2023; Ha & Ivey, 2017). In a community, co‐flowering plant species are often visited by multiple pollinators, resulting in generalized pollination networks (Bascompte et al., 2003). The generalized structure of the pollination networks is thought to buffer against the secondary extinction of species due to environmental perturbations or climate change and is typically studied in the context of mutualistic interactions among species (Bascompte & Jordano, 2007; CaraDonna & Waser, 2020; Vázquez et al., 2009). However, co‐flowering plants may also experience pollination competition (Arceo‐Gómez & Ashman, 2016; Campbell & Motten, 1985). For example, the invasive *Lythrum salicaria* reduced pollinator visits and reproductive success of native congener *L. alatum*, when the two species grew in arrays together (Brown et al., 2002). Plant species may deposit pollen grains on the stigmas of other plants through sharing pollinators, resulting in heterospecific pollen transfer (HPT) (Arceo‐Gómez et al., 2016; Fang & Huang, 2016a).

Heterospecific pollen deposition could occupy stigma area and inhibit conspecific pollen germination (Harder & Barrett, 1992), trigger stigma closure that prevents further conspecific pollen receipt (Galen & Gregory, 1989), or have allelopathic effects (Kanchan & Chandra, 1980). Heterospecific pollen deposition on plant stigmas is generally thought to have negative or neutral effects on plant reproductive success (Ashman & Arceo‐Gómez, 2013; Fang et al., 2023; Morales & Traveset, 2008), although some studies report facilitative effects (Arceo‐Gómez et al., 2019; Streher et al., 2020). Despite the growing number of studies on plant–pollinator mutualism and plant–plant pollination competition, the relationship between these two types of interactions remains poorly understood (Fang & Huang, 2016a, Zhang et al. 2021). The degree of pollinator sharing between plants is expected to influence HPT. For instance, Zhang et al. (2021) found that higher degrees of pollinator sharing and lower flower color dissimilarity between plant species had positive effects on HPT. Moreover, it was found that more abundant and generalized plant species tended to export more pollen to other species, leading to stronger HPT (Arceo‐Gómez et al., 2016; Fang et al., 2023). These findings suggested that generalized plant–pollinator interaction and plant–plant pollen transfer could be interrelated.

Most plant–pollinator networks are constructed based on the observations of flower visits (FV), which record the identity of visitors and visit frequencies (Chacoff et al., 2018; Fang & Huang, 2016b; Vázquez et al., 2005). These observations assume that all visitors are equally effective pollinators. However, a visitor could become an effective pollinator only when it transfers viable pollen from one flower to the stigma of another individual. If a pollinator carries few pollen grains, it may have little effect on the plant's fitness, irrespective of its visit frequency (de Santiago‐Hernández et al., 2019). Pollinators also vary in their capacity to carry and deliver pollen to flowers (Ne'eman et al., 2010). For example, pilosity has been confirmed as a significant determinant of pollen abundance in the body. Fairly furred pollinators carry larger amounts of pollen grains than either poorly or well‐furred pollinator species (Cullen et al., 2021). Therefore, FV networks may not fully reflect the plant–pollinator interactions and interspecific pollination processes. An alternative approach is to construct plant–pollinator networks by identifying the species and amounts of pollen load (PL) on pollinators (Barker & Arceo‐Gómez, 2021; Bosch et al., 2009; de Manincor et al., 2020; Tourbez et al., 2023; Zhao et al., 2019). This approach further explored the effectiveness of visitors as pollen vectors (Zhao et al., 2019), particularly in distinguishing between nectar foraging and pollen collecting, although both are considered effective interactions in FV networks. Moreover, identifying pollen on insect bodies can increase the likelihood of detecting rare plant–pollinator links that are difficult to observe in the field (Alarcón, 2010; Jędrzejewska‐Szmek & Zych, 2013). Previous studies have shown that PL networks observed more diverse links than FV networks, suggesting that pollen load could more accurately capture the plant–pollinator interactions in natural communities (Barker & Arceo‐Gómez, 2021; Bosch et al., 2009; Tourbez et al., 2023). In addition, the PL on the pollinator body can represent interactions between animals and plants over a relatively long period, reducing the constraints of observation time in flower visit network.

Both FV and PL networks imply interactions between co‐flowering plant species. Species pairs with more links in the plant–pollinator network may exhibit a higher degree of pollinator sharing. Recent study revealed that higher pollinator sharing in FV network had positive effects on HPT (Zhang et al., 2021). Since the PL network could be a more complete representation of the plant–pollinator interaction, pollinator sharing that based on PL may better explain interspecific pollination of plants. The emergence of new packages (glmm.hp, Lai et al., 2022) allows us to elucidate the explanatory power of pollinator sharing for interspecific pollen transfer. Exploring the effect of plant–pollinator interactions (FV and PL) on heterospecific pollen transfer may help to understand the relationship between plant–pollinator mutualism and pollination competition. In this study, we investigated the plant–pollinator interaction pattern of a subalpine meadow community in Guizhou Province, Southwest China, and constructed plant–pollinator interaction networks based on flower visit and pollinator pollen load, as well as the heterospecific pollen transfer network. Specifically, we addressed the following two questions: (1) Are there significant differences between the interaction networks of plant–pollinator based on FV and PL? (2) Can the pollinator sharing degree based on PL network better explain the pattern of the heterospecific pollen transfer than the degree based on FV network?

## 2 MATERIALS AND METHODS

### 2.1 Study site and field observation

**FIGURE 1:**
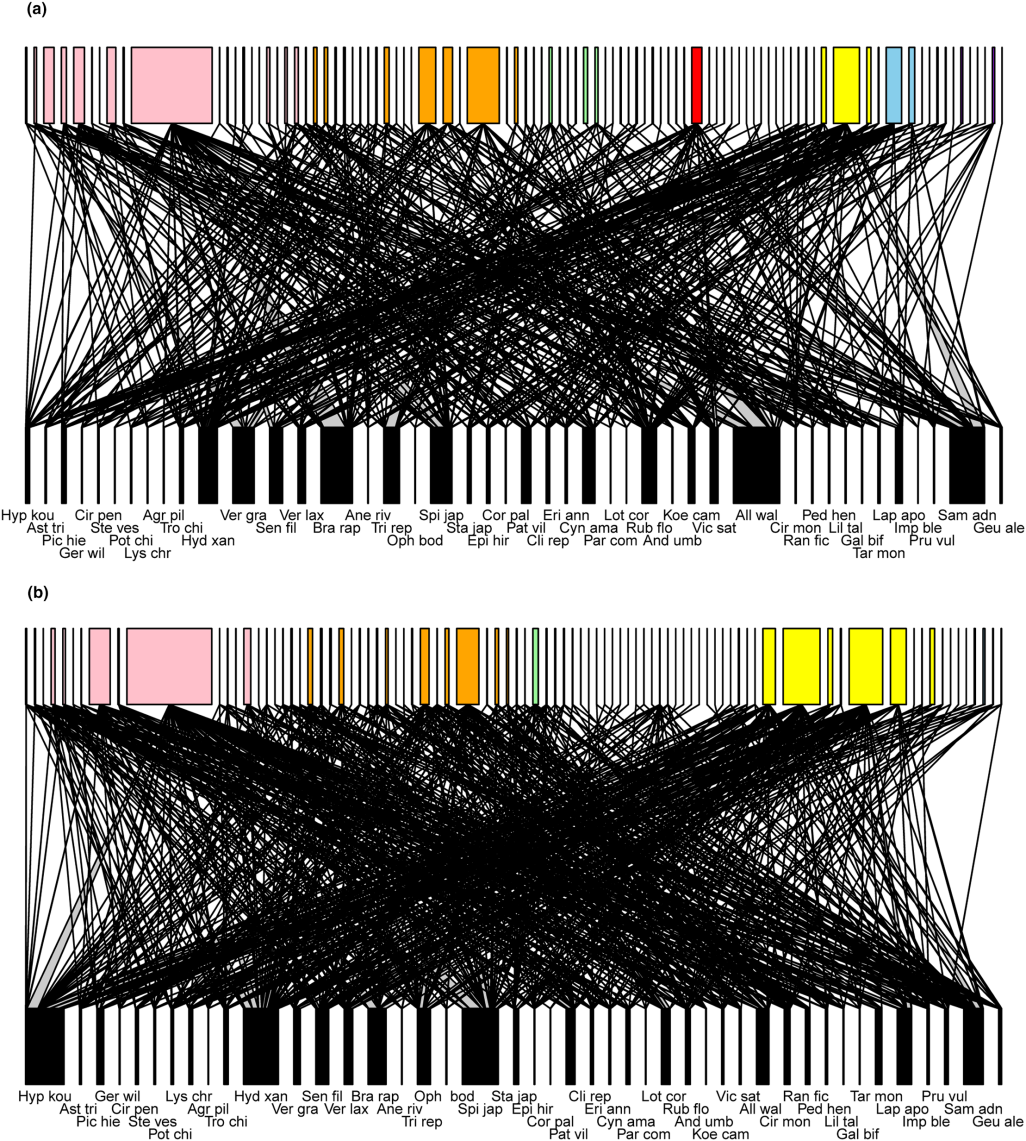
Plant–pollinator interaction networks constructed based on (a) flower (FV) and (b) pollen load (PL) data. Pollinators are represented by nodes on the top (purple, hoverfly; orange, fly; green, bumblebee; red, moth; yellow, butterfly; blue, beetle; purple, bee; black, others) and plants at the bottom. The rectangle width is proportional to the number of interactions recorded per node. The thickness of the lines reflects the frequency of those interactions.

We conducted the research on the southern slope of the Leek Hill Protection Zone in Bijie City, Guizhou Province, Southwest China (104°45′14″ E, 26°59′33″ N, 2640–2730 m above sea level). We surveyed the flowering community during its peak blooming period from July 6th to 22nd in 2022, across a transect approximately 1 km long and 3 m wide (Fang et al., 2023). The floral community was composed of 44 co‐flowering plant species from 24 families (Table S1). We collected pollinator data on clear and calm days. Pollinator visit observation was carried out between 10:00 a.m. and 4:00 p.m., which corresponded to the peak activity period of pollinators at our study site. Each observation lasted for 30 min. We observed each plant at least six times on different days, resulting in a total of 3 h of observation per plant species in 2022. For each census, we observed a similar amount of flowers as possible, about 80–100 flowers, and recorded the pollinator species and the visit amount. For several rare species, such as *Lilium taliense*, the number of flowers per observation is roughly 20–40. We considered visits as effective if the pollinator touched the stamen or stigma of a flower. Flies and hoverflies that visited flowers to search for pollen were also considered pollinators because they could contact the stigma and transfer pollen with their bodies or wings. Unknown pollinators were initially classified based on their morphological characteristics and later identified by experts to the highest possible taxonomic level (see *Acknowledgements*). The FV network was constructed based on visits between plant and pollinator species in the subalpine meadow community (Figure 1a, Table S2).

### 2.2 Pollinator pollen load

During flower visit interaction survey, we collected insects with a sweep net and put each individual into a clean centrifuge tube. Then, the centrifuge tubes were stored in an ice box to restrict the movement and freeze the insects. We used all insect specimens that were collected in a plant species for the pollen load analysis. To construct plant–pollinator network based on the pollen loads on pollinator bodies, we used small pieces of fuchsin gel (3 × 3 × 1 mm) to stick to different body parts of each flower‐visiting insect on the day of the insect caught. The fuchsin jelly was made following a standard protocol, by mixing 175 mL of distilled water with 150 mL of glycerol and 50 g of gelatin, which was then mixed with basic fuchsin crystals. We attached the gel to the top and bottom of the thorax and abdomen, the head and mouth parts, and the antennae and legs of the insect, avoiding the pollen corbiculae of bees. After wiping each insect, the fuchsin gel with pollen samples was placed on a microscope slide and melted over a hot plate. In total, we sampled the pollen loads of all 1086 collected insects. On average, we counted 25.2 ± 15.6 (Mean ± SD) pollinator individuals per plant species. Then, we observed each fuchsin jelly sample under a microscope and identified and counted all the pollen grains in the sample. We used a pollen reference library for all plant species at the study site to identify the pollen grains. A compound light microscope was used at 400× magnification to quantify and identify the pollen grains. If the pollen grain could not be confirmed to match any of the reference species, it was classified as unknown and was excluded from the network (1.1% of total pollen counted). The PL network was constructed based on the pollen species and the relative abundances of each pollinator (Figure 1b, Table S3). The link weight of the PL network represented the pollen amount of one plant species that was carried by one pollinator species.

### 2.3 Pollen transfer network

We collected 30 stigmas from flowers in late anthesis for each plant species across 2 days at the end of observation censuses to ensure that all collected plants have overlapping flowering periods, as well as potential heterospecific pollen transfer. We typically collected one or occasionally two stigmas per plant from 20 to 25 individuals. Each stigma was removed in the field with clean forceps and stored in a microcentrifuge tube with 70% ethanol. Stigma collection was carried out on sunny days to ensure that the pollination was sufficient. In the laboratory, we placed the stigmas on glass slides and squashed them with a cover glass, then photographed the pollen grains with a digital camera attached to a microscope at 400× magnification. Then, we used Image‐Pro Plus 5.0 to measure the shape characteristics of pollen grains. To identify the pollen grains, we compared all pollen grains with a pollen reference library using morphological features including size, shape, darkness, and exine ornamentation. The library was created based on pollen grains obtained from adjacent flowers collected in the field. To construct the heterospecific pollen transfer (hereafter HPT) network, we counted the number of pollen grains transferred from donor to recipient species, following Zhang et al. (2021). In the case of HPT, the link value counts the number of pollen grains transferred from donor species to recipient species.

### 2.4 Statistical analyses

To elucidate the overall difference between FV and PL networks, we first compared the two networks using procrustes analysis (Alarcón, 2010; Barker & Arceo‐Gómez, 2021). Procrustes analysis is a method for relating two sets of multivariate observations, which could evaluate the differences in network shape based on corresponding landmarks (plant or pollinator species) within the networks. Significant differences in procrustes analysis could indicate structural differences between the two networks. Then, to test whether species had similar positions in the two networks, we compared several species‐level indices by Pearson correlation, including Betweenness (the centrality of a species by its position on the shortest paths between other species, ranging from 0 to 1), Closeness (the centrality of a species by its path lengths to other species, ranging from 0 to 1), and *d*′ (specialization of each species based on its discrimination from random selection of partners, ranging from 0 to 1). We also performed rarefaction analysis using the iNEXT package in R 4.2.3, which showed that our sampling effort captured the majority of interaction diversity for the flower visit, the pollen load, and the HPT networks (Figure S1).

To investigate the effect of flower visit (FV) and pollen load (PL) networks on heterospecific pollen transfer, we first transformed the plant–pollinator interactions into potential plant–plant interactions by calculating Müller's index, ranging from 0 (no pollinator sharing) to 1 (same visitation spectrum), as a proxy of how much one donor species contributes to the diet of all pollinators shared with another recipient species (Carvalheiro et al., 2014; Müller et al., 1999; Zhang et al., 2021). Then, we employed a generalized linear mixed model (GLMM) with a Poisson error distribution and a log‐link function using Müller's indices derived from the FV and PL networks as fixed effects, and donor and recipient species involved in pollen transfer as random effects. In order to further elucidate the individual contributions of the FV and PL in driving the effect size of HPT, we conducted hierarchical variance partitioning analysis, which could calculate individual contributions of each predictor (fixed effects) toward marginal *R* ^2^ for generalized linear mixed‐effect model. Müller index was calculated with the bipartite package in R (ver. 4.2.3). The GLMM statistical analyses were carried out with R (ver. 4.2.3) using the lme4 (Bates et al., 2014) package and the glmm.hp package (Lai et al., 2022).

## 3 RESULTS

In total, the flower visit network consisted of 14,560 visits and 440 links between 92 pollinator species and 44 plant species (Figure 1a, Table 1), in which each plant species was visited by 10.0 ± 4.8 (mean ± SD) pollinator species and received 330.6 ± 482.0 visits on average. Among the plant species, *Rubus parvifolius* and *Hydrangea aspera* had the most pollinator species (23 pollinator species, 26.2% of total). *Allium wallichii* received the most visits, accounting for 14.9% of the total.

**TABLE 1:** Network properties of the flower visit and pollen load networks in a subalpine meadow community in the Leek Hill Protection Zone in 2022.

Statistic	Flower visit	Pollen load
No. plant species	44	44
No. insect visitor species	92	86
No. interactions	440	643
Connectance	0.11	0.17
Link per species	3.24	4.95
Link diversity	6.93	8.77
Interaction evenness	0.58	0.60
H′2	0.46*	0.37*
NODF	21.38*	37.04*
wNODF	13.18*	25.02*

* *p* < .01. The asterisks represent statistically significant network metrics that did not overlap with those of null model expectations.

Among the 1086 insect individuals collected and identified, Hymenoptera and Diptera accounted for 33.9% and 54.2%, respectively. Lepidoptera and Coleoptera represented 9.8% and 1.8%, respectively. On average, each pollinator species captured 11.8 individuals. Among the collected insects, there were 185 individuals that did not carry any pollen load. We identified the pollen load of the remaining 901 pollinators, which belonged to 86 species. Each pollinator body carried an average of 168.7 ± 319.9 pollen grains from 1.8 ± 0.8 plant species. The PL network included 165,646 pollen grains and 643 links between 86 pollinator species and 44 plant species (Figure 1b, Table 1), in which each plant species was linked with an average of 14.6 ± 7.5 pollinator species. There were 30.8% of pollinator species that carried pollen from *Hypericum monogynum*, accounting for 12.4% of the total pollen load on insect bodies.

**FIGURE 2:**
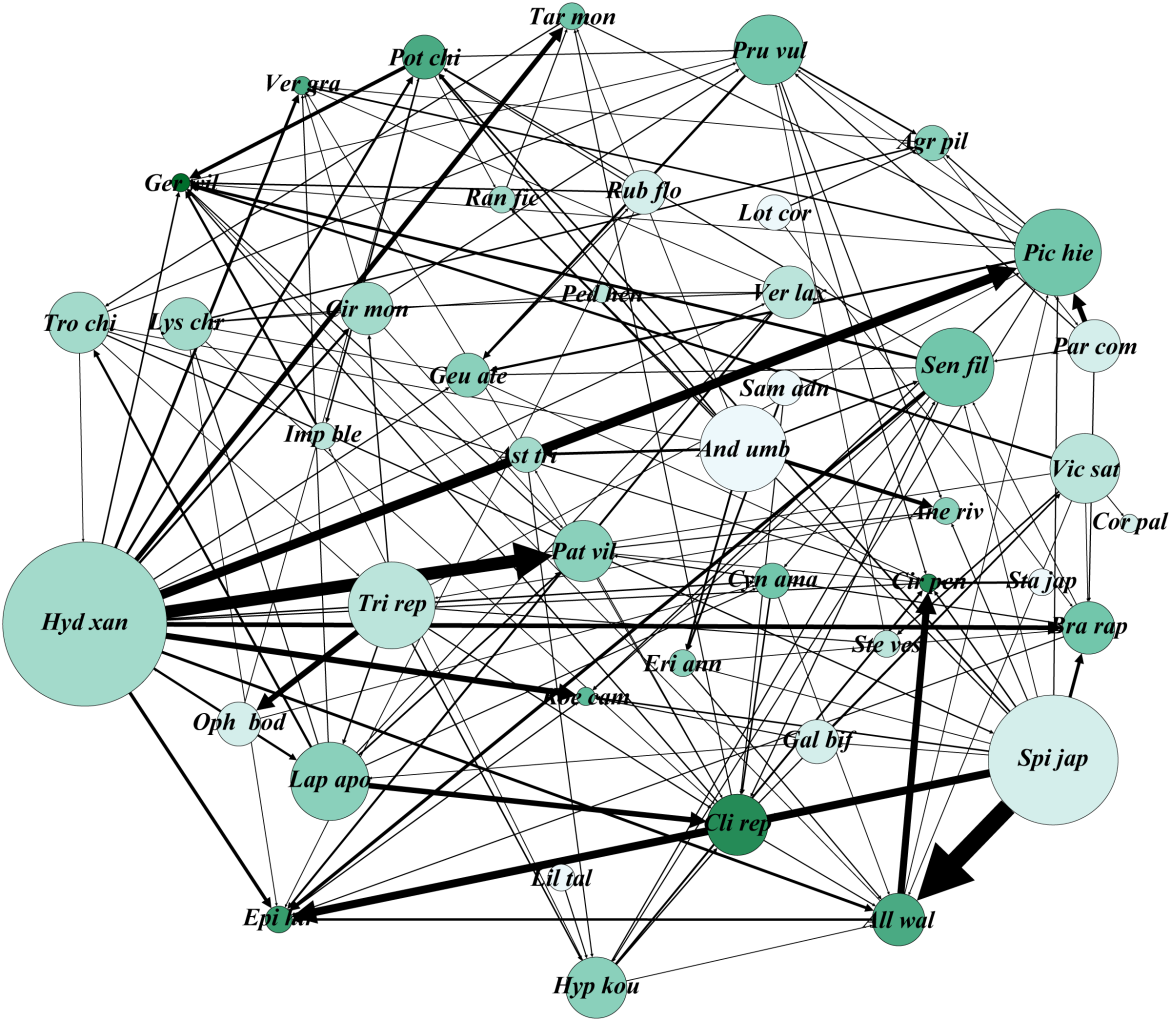
The directed network of heterospecific pollen transfer among 44 plant species. Heterospecific pollen transfer is shown as links between nodes, and the arrow marks the direction of pollen transfer from the donor to the recipient. Node size represents exported species (Out degree), with larger circles representing species that exported HP to more species. Node darkness represents imported species (In degree), with darker color indicating that species received HP from more plant species. Species abbreviations are given in supporting information (Table S4).

A total of 44,900 conspecific and 1171 heterospecific pollen grains were identified on 1320 stigmas from 44 plant species. The HPT network included 158 links, with an average link strength of 7.4 ± 14.5 (Figure 2). In HPT network, *H. aspera* exported the largest amount of heterospecific pollen to the most diverse partners (29.2% of total HP amount, 17 plant species), while *A. wallichii* received the largest amount of heterospecific pollen (12.3% of the total HP amount) and *Geranium wilfordii* received the most diverse HP species (11 species). Five plant species received no heterospecific pollen on stigmas (*Lotus corniculatus*, *Stachys japonica*, *Androsace umbellata*, *Lilium taliense*, and *Sambucus adnata*), and six plant species transferred no heterospecific pollen (*Cirsium pendulum*, *Corydalis linarioides*, *Geranium wilfordii*, *Pedicularis henryi*, *Veratrum grandiflorum*, and *Koenigia campanulata*).

### 3.1 Comparison between FV and PL networks

Flower visit and pollen load networks exhibited a high degree of overlap. The overlapping links contributed 70.4% and 48.1% of total links in the FV and PL networks, accounting for 89.3% and 81.1% of total visits and pollen grains, respectively. Null model analyses showed that all network structure properties were significantly different from what would be expected by random (all *p* < .01, Table 1). The Betweenness, Closeness, and *d*′ of plant and pollinator species were positively related in the two networks (Table 2), indicating that the plant and pollinator species had similar positions in the FV and PL networks. However, the shape of the FV network was significantly different from that of the PL network (Euclidean distance, Procrustes correlation = .35, sum of squares = 0.88, *p* = .01).

**TABLE 2:** Results of Pearson correlation of Betweenness, Closeness, and *d*′ for the plant and pollinator species between flower visit and pollen load network.

	Plant (*n* = 44)		Pollinator (*n* = 86)	
	*r*	*p*	*r*	*p*
Betweenness	.40	.007	.83	<.001
Closeness	.44	.003	.75	<.001
*d*′	.42	.004	.29	<.001

### 3.2 The effect of flower visitor and pollen load networks on HPT

In the FV network, 87.4% of pairs of plant species exhibited varying degrees of pollinator sharing, with Müller's index ranging from 0 to 0.41. In the PL network, pollinator sharing was more prevalent between pairs of plant species, reaching 98.8%, with Müller's index ranging from 0 to 0.30. The Müller's indices were not significantly different between the FV and PL networks (*T* test, *t* = −0.90, *p* = .37, df = 3782; Levene test, *F* = 0.22, *p* = .64). There was a positive correlation between Müller's index derived from the FV and PL networks (Pearson correlation, *r* = .41, *p* < .001, *n* = 1892). The GLMM results revealed a significant positive effect of pollinator sharing degrees that derived from both the FV and PL network on HPT (FV, *Z* = 6.423, *p* < .01; PL, *Z* = 3.908, *p* < .01; Table 3a). However, the hierarchical variance partitioning analysis indicated that the explanatory power of fixed factors was weak, which explained about 1.8% of the variance of the effect size of HPT (Table 3a). The pollinator‐sharing degrees derived from the two networks exhibited almost equal performances in explaining the variance of HPT (FV, *R* ^2^ = .009; PL, *R* ^2^ = .009). Then, we recalculated the data for plant pairs that observed HPT (all cases where HPT > 0). The explanatory power of fixed factors was improved, which explained about 8.1% of the effect size of HPT (Table 3b).

**TABLE 3:** Results of generalized linear mixing model with Poisson distribution for HPT and hierarchical variance partitioning, in total 1892 observations.

(A) Formula	Effects	Coefficient	SE	*p* value
HPT ~ FV + PL + (1|donor) + (1|recipient)	(Intercept)	−2.981	0.356	<.001
	Flower visit	6.423	0.707	<.001
	Pollen load	3.908	1.131	<.001
**Hierarchical variance partitioning**	**Marginal *R*** ^**2**^	**FV (Part *R*** ^**2**^ **)**	**PL (Part *R*** ^**2**^ **)**	**Conditional *R*** ^**2**^
Delta	.017	.009 (52.1%)	.008 (47.9%)	.828
Lognormal	.018	.009 (52.0%)	.009 (48.0%)	.876
Trigamma	.015	.008 (52.0%)	.007 (48.1%)	.739

(B) Formula	Effects	Coefficient	SE	*p* value
HPT1 ~ FV + PL + (1|donor) + (1|recipient)	(Intercept)	0.765	0.210	<.001
	Flower visit	6.365	0.901	<.001
	Pollen load	2.718	1.612	.09
**Hierarchical variance partitioning**	**Marginal *R*** ^**2**^	**FV (Part *R*** ^**2**^ **)**	**PL (Part *R*** ^**2**^ **)**	**Conditional *R*** ^**2**^
Delta	.081	.037 (45.1%)	.044 (54.8%)	.897
Lognormal	.082	.037 (45.2%)	.045 (54.8%)	.905
Trigamma	.080	.036 (45.3%)	.044 (54.7%)	.888

*Note*: FV (flower visit) and PL (pollen load) indicated the pollinator sharing degree (Müller's index) that was derived from flower visit and pollen load networks. HPT1, all cases where HPT > 0, in total 158 observations.

## 4 DISCUSSION

The quality and quantity of pollen deposited on stigmas are the final consequences of the pollination process. Heterospecific pollen transfer caused by shared pollinators among plant species may affect plant reproduction (Morales & Traveset, 2008). Our results show that the degree of pollinator sharing, based on either the FV or PL network, is positively related to the amount of heterospecific pollen transfer between plant species pairs. The explanatory power of the degree of pollinator sharing, derived from either the FV or PL network, accounted for a small proportion of the variance in HPT. In addition, the explanatory power was similar in both the FV and PL networks.

In our study, the FV network was more likely to be a subset of the PL networks, and most links in the FV network are confirmed in the PL network, but not vice versa. The pollen load network observed more links and could describe the plant–pollinator interactions of the community more completely. For example, the number of links in the PL network was 46% more than that in the FV network, and the number of partner species per plant species increased from 10.0 ± 4.9 in the FV network to 14.6 ± 7.5 in the PL network. The unique links of each network generally had lower link strength, indicating that the PL could detect more links. These results are similar to previous studies (Barker & Arceo‐Gómez, 2021; Bosch et al., 2009; Tourbez et al., 2023), which found that networks based on pollen load increased the connectivity and the average degree of plants and pollinators, and reduced the specialization of the networks. We also found that the positions of plant and pollinator species in the FV and PL networks were significantly correlated, indicating that both network types are effective in identifying important plant–pollinator interactions from different perspectives. For instance, de Manincor et al. (2020) compared FV and PL networks at three sites and found no significant difference in the specialization index *d*′ between the two networks. In addition, a recent study using procrustes analysis found no significant difference in the overall structure of FV and PL networks (Barker & Arceo‐Gómez, 2021). However, in our study, we found significant structural differences between the two networks. This may be due to the great differences in body pollen load among pollinator functional groups, resulting in a large difference in link strength distribution between PL and FV networks (Zhao et al., 2019). For example, in a study that investigated the abundance, diversity, or composition of pollen carried by pollinators, the pollen load and diversity were suggested to be positively correlated with body size of the pollinator, and bees carried more diverse pollen load than flies (Cullen et al., 2021). In our study, bees and flies, both the major pollinator groups in our community, differed greatly in pollen load capacity. The two pollinator groups comprised 14.6% and 23.5% of the total captured individuals, respectively, but each bee carried about 231 pollen grains, while each fly carried only 68 pollen grains. Moreover, a plant–pollinator link may have a high visitation in the FV network, but a small pollen load on the pollinator. Or, it may have a low visitation in the FV network, but a large pollen load on the pollinator. This mismatch in link strength may cause the structural difference between the two networks in procrustes analysis. Overall, our results suggested that both the FV and PL networks can observe the main plant–pollinator interactions in natural communities.

In generalized pollination networks, plants commonly share pollinators. We found prevalent pollinator sharing, with a prevalence of 87.4% based on the FV and 98.8% based on the PL network. However, only about 10% of plant species pairs had HPT, which suggested that pollinator sharing did not necessarily result in heterospecific pollen transfer. A previous study revealed that the pollinator sharing based on FV network had positive effects on HPT (Zhang et al., 2021), but did not evaluate the explanatory power. In this study, we confirmed the positive effect of pollinator sharing on HPT, but in low explanatory power, only about 2% of the HPT amount was explained. In the model where HPT amount was greater than 0, the fixed factors explained 8% of the HPT amount. One possibility for low explanatory power is that pollination networks described a snapshot of plant–pollinator interactions that varied substantially in time, within days, seasons, or across years (Chacoff et al., 2018; Fang & Huang, 2016a; Fründ et al., 2011; Petanidou et al., 2008). The amount and the type of pollen that accumulated on the stigmas were the outcomes of several days of pollination. Multiple layers of snapshots may better explain the HPT amount. Several studies have shown that the pollinator interaction networks of FV and PL exhibit similar characteristics and can represent the main processes of plant–pollinator interactions (Barker & Arceo‐Gómez, 2021; Tourbez et al., 2023). Therefore, the degree of pollinator sharing in both FV and PL networks should reflect the potential pollination interference between plant species. The degree of pollinator sharing based on the FV and PL had almost equal explanatory power, which also mirrored that both networks observed the main plant–pollinator interactions. Another explanation is that flower visits and pollinator pollen load could not fully reflect the pollination efficiency (Ne'eman et al., 2010). Flower characteristics, such as the position of male and female functions, and pollinator characteristics, such as pollinator pilosity and grooming behavior, may both affect pollen transfer (Zhao et al., 2019). In our model, random effects of HPT donor and recipient species contributed to large explanatory power. This suggests that differences between donor and recipient plant species, especially the traits related to pollen export and pollen deposition, maybe the major factors affecting pollen transfer (Ashman & Arceo‐Gómez, 2013; Minnaar et al., 2019). For example, a recent study that investigated how plant–pollinator interactions affect heterospecific pollen transfer in a biodiversity hotspot found that larger stigmas, longer styles, and more exposure stigmas could lead to higher receipt of HP (Wei et al., 2021). Therefore, it is necessary to take into account the dissimilarity of flower characteristics between the donor and the receipt species, which may better explain the pollen transfer among co‐flowering species.

Our study provides valuable insights into the structure of plant–pollinator interaction networks based on the pollinator visit and the pollinator pollen load, and effects of the FV and PL network on heterospecific pollen transfer among plant species in a subalpine meadow. Our results highlight that both the flower visit and the pollen load observed the major plant–pollinator interactions, and described interactions from different perspectives. Pollen load network observed more links, and flower visit network tended to be a subset of pollen load network. The degree of pollinator sharing was positively correlated with heterospecific pollen amount but explained only a small portion of the variation. Future studies should investigate the potential trait match that affects heterospecific pollen transfer and reproductive success among donor and recipient combinations (Arceo‐Gómez et al., 2019; Streher et al., 2020; Suárez‐Mariño et al., 2019), which would increase our understanding of the intermediate stages in the pollination process, as well as the ecological and evolutionary consequences of pollinator sharing and heterospecific pollen transfer.

## AUTHOR CONTRIBUTIONS

**Qiang Fang:** Conceptualization (lead); funding acquisition (lead); methodology (lead); project administration (supporting); writing – original draft (lead); writing – review and editing (equal). **Jiajun Wu:** Data curation (equal); formal analysis (supporting); investigation (equal); methodology (equal); project administration (equal); writing – original draft (supporting); writing – review and editing (equal). **Tao Zhang:** Conceptualization (supporting); investigation (supporting); methodology (equal); project administration (supporting); writing – original draft (equal); writing – review and editing (equal). **Suyan Ba:** Data curation (equal); investigation (equal); methodology (equal); writing – review and editing (supporting). **Chunyan Zhao:** Data curation (equal); investigation (equal); project administration (equal); writing – review and editing (supporting). **Panfeng Dai:** Conceptualization (supporting); investigation (supporting); project administration (supporting); writing – original draft (supporting); writing – review and editing (supporting).

## CONFLICT OF INTEREST STATEMENT

The authors declare no conflicts of interest.

## Supporting information


Figure S1

Tables S1–S4

## Data Availability

All data are provided as supporting information.
