# Supplementary figures and images for: Flower visits and pollinator pollen load networks reveal the effects of pollinator sharing on heterospecific pollen transfer in a subalpine plant community
Source: Ecol Evol. 2024 Apr 8;14(4):e11244. doi: 10.1002/ece3.11244 (PMC10999945; doi:10.1002/ece3.11244)

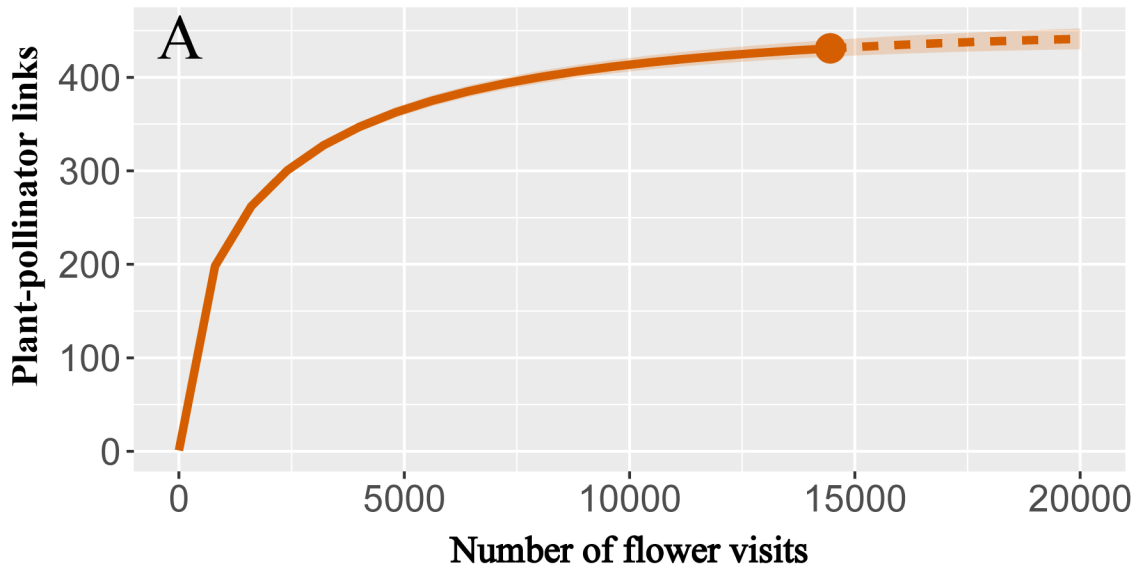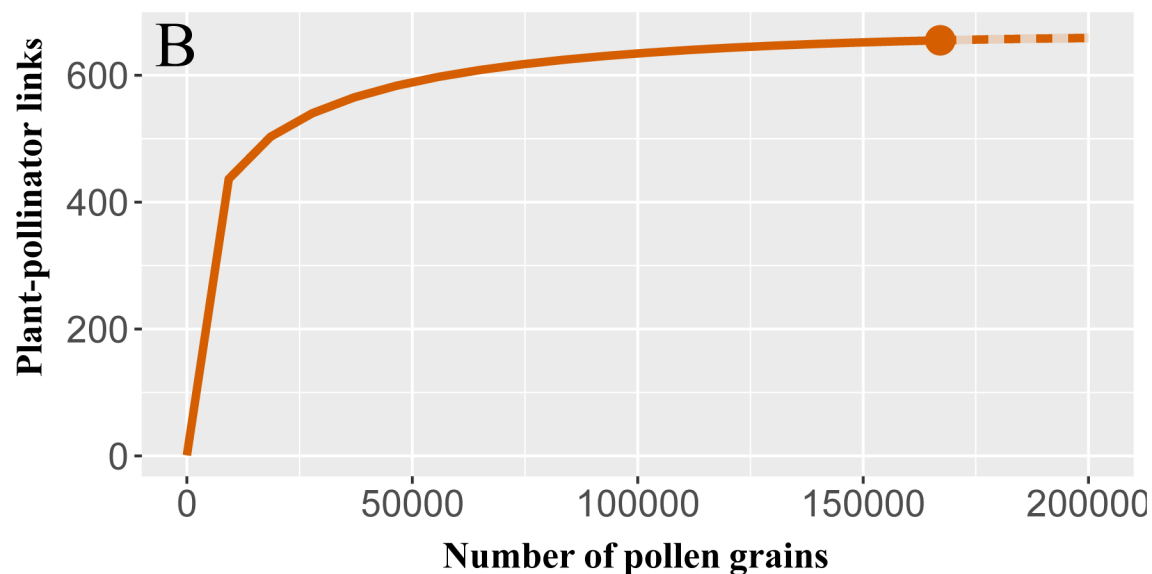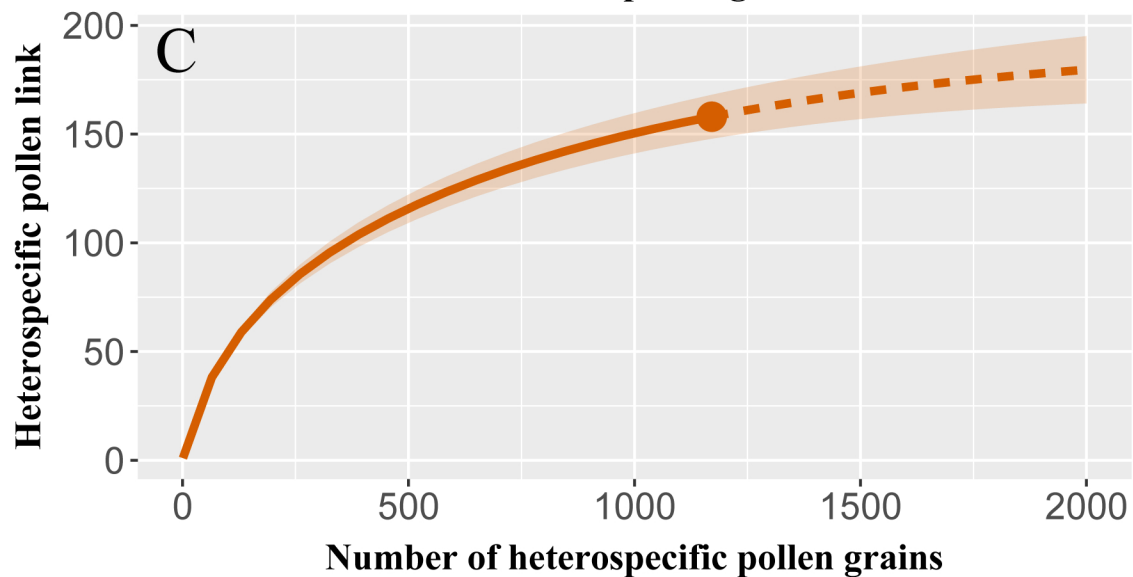

Supplement: Supplementary file 1 — Figure S1 [file ECE3-14-e11244-s002.pdf]
